# Survey of practitioners’ competency for diagnosis of acute diseases manifest on chest X-ray

**DOI:** 10.1186/s12880-017-0222-8

**Published:** 2017-08-18

**Authors:** Ghazaleh Mehdipoor, Fatemeh Salmani, Abbas Arjmand Shabestari

**Affiliations:** 1 Department of Radiology, Modarres Hospital, Shahid Beheshti University of Medical Sciences, Tehran, Iran; 2 Department of Biostatistics, School of Allied Medical Sciences, Shahid Beheshti University of Medical Sciences, Tehran, Iran

**Keywords:** Chest X-ray, Competency, Knowledge, Diagnosis

## Abstract

**Background:**

Chest X-ray (CXR) is a common imaging modality that could impact immediate decision-making for acute chest pathologies. We sought to examine the non-radiologists proficiency of diagnosing acute pathologies manifest on CXR.

**Methods:**

We selected 9 clinical vignettes, each associated with a CXR, wherein only a single acute chest pathology was manifest. We also added a low-risk vignette associated with a normal CXR. We built an electronic survey with the CXR-embedded vignettes and also inquired about the participants’ confidence in the diagnosis, and prior exposure to the topics. We distributed the survey to senior medical students and general practitioners (GPs) in Tehran, Iran. We scored each correct answer per each vignette as 1 and each incorrect answer as 0; leading into a sum score from 0 to10 for the entire survey for each participant.

**Results:**

Of the 136 candidates, 100 had legible survey results (67 medical students and 33 GPs). The overall score (mean [standard error]) was 3.57 [0.20], with no significant difference between the students and GPs (*P* = 0.15). The lowest rate of correct response occurred for acute respiratory distress syndrome (8%), foreign body (12%), and normal CXR (15%), while the best-answered vignettes were diaphragmatic herniation (77%) and pneumoperitoneum (67%). Self-reported confidence was associated with correct response for pneumoperitoneum, tension pneumothorax, and pulmonary edema (*P* < 0.05 for all).

**Conclusions:**

Diagnostic proficiency of practitioners for acute chest pathologies in our study was poor, including for distinction of a normal CXR. Such dramatic knowledge deficiencies for common or life-threatening chest pathologies should be prioritized in the educational and continuous education curricula. Secure electronic tools for transferring the CXRs to specialists in case of acute pathologies would be an interim pragmatic alternative.

**Electronic supplementary material:**

The online version of this article (doi:10.1186/s12880-017-0222-8) contains supplementary material, which is available to authorized users.

## Background

Dyspnea and chest pain are among the most common presentations of thoracic diseases in emergency departments and office visits [1, 2]. Chest X-ray (CXR) is a practical, inexpensive, and widely available imaging modality that plays an integral role in the evaluation of thoracic pathologies, primarily of cardiopulmonary origin. Appropriate interpretation of CXR findings commonly impact clinical decision-making and management strategies [3]. In many acute situations, there is an immediate need to make point-of-care interpretations of the CXRs to guide further testing [4], triage the level of care [5], or make acute therapeutic interventions [3, 6].

Although common sense and multiple studies show that radiologists provide more accurate interpretation of CXRs, other providers should also be cognizant of acute CXR pathologies. Some prior studies indicate that medical students, and other junior practitioners, may be less familiar with or confident of their knowledge about detection of CXR findings such as pulmonary metastases, pleural effusion, tuberculosis, and dextrocardia compared with senior providers and radiologists [7–14]. Such subacute or chronic pathologies, while important, might not necessarily need urgent attention, and hence, clinicians can wait for formal interpretation by radiologists. However, acute pathologies may require immediate interpretation for subsequent decision-making, which commonly precedes a formal conventional review by radiologists. It is unknown to what extent potential knowledge deficiency or experience gap exists for diagnosing the CXR findings in acute chest pathologies, such as in tension pneumothorax, aortic dissection, or more common scenarios such as pulmonary edema, wherein appropriate interpretation impacts immediate plan of care. We aimed to assess the competency of non-radiologist healthcare providers (senior medical students who participate in point-of-care clinical decisions, and general practitioners (GPs)) for diagnoses of acute life-threatening conditions manifest on CXRs. We also determined the factors associated with increased odds of accurate radiographic interpretations by such providers.

## Methods

### Setting and participants

The survey was conducted between January 20, and February 30, 2015. We surveyed two subsets of participants whose clinical interpretations could impact clinical decision-making: First, we included medical students in the last (7th) year of training, who were assigned to pre-graduate internship rotations. All participants had prior training didactics for radiology during clinical clerkship rotations. Second, we also included community GPs who were practicing independently. We excluded recent graduates who were not working as permanent GPs. Most of such physicians will opt to pursue graduate medical education in near future, and many are actively getting prepared for residency selection examinations, thereby not representing a cohort of participants with expectably stable knowledge base.

### Survey preparation and piloting

**Fig. 1:**
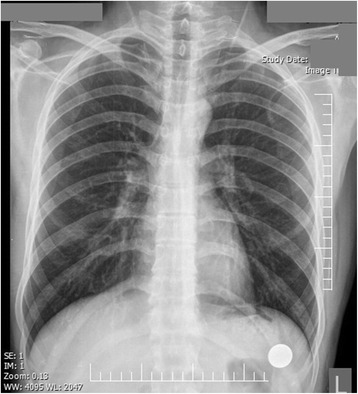
A patient complaining of intermittent dyspnea and chest pain. Normal. Image source: Shahid Beheshti University of Medical Sciences

**Fig. 2:**
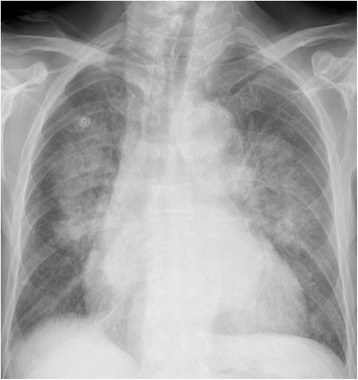
A 65 year-old man with remote history of myocardial infarction presenting with worsening dyspnea, shortness of breath and progressive respiratory distress. The CXR demonstrates bilateral pulmonary hilar vascular engorgement resulting in bats wing appearance accompanied by less perceptible prominence of pulmonary vascular markings through the peripheral zones and presence of cardiomegaly. Acute pulmonary edema. Available at: *http://chestatlas.com/gallery/main.php?g2_itemId=1105*. Reproduced with permission from *Chest Atlas*

**Fig. 3:**
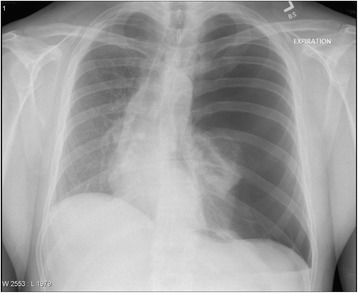
A 29 year-old man with severe respiratory distress and hypotension in the setting of persistent left hemithorax pain following a cough attack. The CXR demonstrates loss of pulmonary vascular markings on the left side, leading to its remarkable left hemithoracic translucency associated with left pulmonary total collapse and rightward mediastinal shift. Tension pneumothorax. Available at: http://chestatlas.com/gallery/main.php?g2_itemId=534. Reproduced with permission from *Chest Atlas*

After discussions between the first and senior author, and per consideration of commonness and clinical importance, we chose 9 CXR diagnoses that represented acute medical or surgical chest pathologies (see Table 1). Next, we made an extensive search for copyright-eligible (i.e. open-access) images representing each of those diagnoses. We made every effort to find images wherein the CXR findings were diagnostic of only one acute chest pathology per image and excluded images with coexisting conditions, normal variants, artifacts or other major differential diagnoses. Subsequently, to make those images more relevant to real-world practice, we designed –on our own and not from any true patient story –clinical vignettes to go along with each image. A tenth low-risk vignette along with a normal CXR was also included (see Figs. 1, 2 and 3 for select examples of vignettes, and Table 1 for all clinical diagnoses in the 10 cases). Selected images were reviewed and confirmed by two authors (GM and AAS). We used Microsoft Excel 2013 for Windows, (Microsoft Corporation, Redmond, WA) to embed the images and build the survey. The survey included questions about the diagnosis, self-reported confidence in the diagnosis, self-reported prior exposure to the presented conditions for all participants, and year of graduation for GPs. The choices of answers included naming the diagnosis by free-texting the answer, or selecting “normal”, or “I don’t know” options. We had specifically opted for free-texting the answers, rather than providing multiple choice answers, to replicate real-world practice and to avoid providing a hint to participants [15, 16]. We measured the participants’ level of confidence for their answers to each vignette with a 5-point Likert scale (higher score indicating greater self-reported certainty in the answer provided). In addition, they also had the option to report their familiarity with (i.e. prior exposure to) similar images for each case with Yes/ No options.

**Table 1:** List of the All Diagnoses in the 10 Vignettes and Response Data per Each Vignette

	Correct (%)	Correct and Certain (%)	Prior Exposure (%)
Aortic Dissection	24	22	13
ARDS^a^	8	7	22
Diaphragmatic Herniation	77	50	31
Foreign Body	12	9	30
Normal	15	13	44
Pericardial Effusion	27	19	62
Pneumoperitoneum	67	23	68
Pneumothorax	54	19	54
Pulmonary Edema	40	31	45
Tension Pneumothorax	33	18	45

^a^ *ARDS* acute respiratory distress syndrome

The electronic survey was piloted by 3 radiology residents and 6 medical students under the same conditions as the main study. Piloting the survey among the radiology residents was specifically helpful to ascertain that each of the selected CXRs would not represent more than a single pathology, and among medical students to ascertain that they were comfortable with the survey platform and vignettes associated with each image. There was 100% consistency in the diagnoses between radiology residents and the designated diagnoses of selected images.

### Survey dissemination

The survey was run in-person by one of the authors (GM) by visits to each of the main teaching facilities of Shahid Beheshti University of Medical Sciences, one of the largest medical schools in Tehran, Iran. A brief in-person introduction, as well as instructions were provided to each participant. Participants were made aware that the results would be used for academic purposes, including publications. Each participants completed the survey on the same laptop computer independently, in order to keep the conditions and test quality consistent for all participants. Per each vignette, each correct answer was scored as 1, and each incorrect answer was scored as 0, leading into a total score from 0 to 10 for the entire survey for each participant. Vague free-texted answers and discrepancies were discussed between two authors (GM and AAS) to reach consensus for appropriate scores.

No patient identifiers were used in any parts of the survey or the resultant manuscript. The associated vignettes did not belong to real patients and were drafted by the authors to fit along with the CXRs. The participants (students and GPs) gave verbal informed consent for use of the data for academic purposes. It is not mandatory or routine practice in Iran to obtain Institutional Review Board approval for surveys of practitioners. The survey participants were aware of the data being used for academic purposes. As such, no ethics committee approval was required for the study.

## Statistical analysis

We assessed the frequency of correct answers for each vignette and the total score of each participant. Total scores were presented as mean and standard error of the mean (SEM) and we used frequencies for the categorical variables (rate of correct responses per each vignette, degree of certainty, self-reported prior exposure to the topics). We used the chi-square test to determine the association between categorical variables (self-reported prior exposure and correctness of diagnosis for each vignette), and the t-test for comparison of the means of total score between the two groups of medical students and GPs. For years passed since the graduation of GPs and their total score, we used Pearson’s correlation. Where the data were non-parametric, we used non-parametric equivalents (Spearman’s test to determine the correlation between certainty and correctness of diagnosis for each vignette).

In order to study the impact of participants’ self-awareness of their knowledge (or lack thereof) in diagnoses of acute CXR findings, we generated a new variable using the categories related to self-reported certainty. For that analysis, being certain (Likert scale 4 or 5) of the correct diagnosis and making the correct diagnosis; or alternatively, self-reported uncertainty (Likert scale 1 or 2) of the correct answer and making subsequent incorrect diagnoses were both scored as 1. In contrast, discordance between the correctness of diagnosis and self-reported certainly led to a score of 0 for each vignette. For this analysis, we excluded the ones with certainty score of 3 on the Likert scale. We used a two-sided *P*-value of <0.05 for all analyses. The data were analyzed using SPSS Version 17.0 (SPSS. Inc., Chicago, IL, USA).

## Results

We distributed the survey to 136 participants (76 medical students and 60 GPs), of whom 105 agreed to participate (response rate: 77%) and 100 had legibly completed data. Of those 100 participants, 67 were senior medical students, and 33 were independently-practicing GPs.

### Overall score and response data per each vignette

The overall score (mean, [SEM]) achieved by all participants in our survey was 3.57, [0.20]. The overall score among medical students and GPs were 3.77, [0.25] and 3.15, [0.33], respectively, with the maximum score being 8 in both groups. The overall score was not significantly different between the two groups (*P* = 0.15).

Overall, the best-answered vignettes were diaphragmatic herniation (77%), pneumoperitoneum (67%), and pneumothorax (54%), while the lowest rate of correct responses occurred for acute respiratory distress syndrome (ARDS) (8%), foreign body (12%), and the low-risk vignette associated with the normal CXR (15%) (Table 1).

### Certainty and awareness of own responses

The participants expressed greatest degree of certainty –defined as certainty scores of 5 on a Likert scale– for pneumoperitoneum (46.9%) and pneumothorax (41.8%) while the greatest degree of uncertainty belonged to ARDS (24.7%) and foreign body (19.3%) (Likert scale: 1). The degree of self-reported certainty in the diagnosis was associated with the correctness of diagnosis for pneumoperitoneum (*P* < 0.001), tension pneumothorax (*P* < 0.001), and pulmonary edema (*P* < 0.05) but not for other conditions.

### Self-reported prior exposure to the topic

The participants reported prior exposure to a similar image most commonly for pneumoperitoneum (68%), and pneumothorax (54%); two images that happened to be among the best-answered vignettes. The normal CXR was claimed to be seen before by only less than half (44%) of the participants.

We observed an association between self-reported exposure to similar images in the past and correctness of diagnosis, for pneumoperitoneum, pericardial effusion, and pulmonary edema (*P* < 0.05 for all) but not for other conditions.

### Self-awareness of One’s performance

Overall, there were 933 observations (evaluable vignettes from the 100 participants; a few of whom had missing values for certainties for some of the 10 vignettes). Of those, 211 observations were excluded because of intermediate certainty (Likert score of 3). Therefore, for this analysis, 722 observations were utilized. Overall, 428 observations (59.2%) showed insight about their answers (i.e. being certain and giving a correct answer or having a high degree of uncertainty and giving an incorrect answer).

### Year of graduation

The year passed from the graduation of GPs ranged from 2 to 25 years and had normal distribution. We observed negative correlation between the years passed from graduation among GPs and total score (*r* = −0.44, *P* = 0.01).

## Discussion

In our study, most of the participants failed to correctly diagnose the majority of acute pathologies with diagnostic clues on CXR. Further, the vast majority (85%) had trouble identifying the normal CXR as being normal, and only less than half reported to have seen it before. Surprisingly, many participants reported to have been certain of a diagnosis when their designated diagnosis was, in fact, incorrect; what could ultimately impact clinical decision-making. Only 59% had appropriate self-perception about the correctness or incorrectness of their answers, as reflected by their self-reported certainty in their answers. The results were consistent among senior medical students and GPs, alike.

Timely detection of acute chest pathologies –particularly the ones reflected in our clinical vignettes –are of paramount importance, especially because appropriate interventions could impact the clinical course and outcomes; as is the case with the use of the electrocardiogram for detection of pathologies such as acute myocardial ischemia in the appropriate clinical setting. Our findings, of very poor diagnostic competency of both junior (student) and independent (GP) practitioners, is highly concerning, since missing diagnoses such as tension pneumothorax, or even more common pathologies such as acute pulmonary edema, could have life-threatening detrimental consequences. Similarly, frequent misdiagnosis of a normal CXR in a low-risk clinical vignette as various pathologies (such as obstructive airway disease with or without superimposed pneumonia), would be concerning for inappropriate additional diagnostic studies or unnecessary (and potentially harmful) therapeutic interventions. Our findings are particularly important, given that the increasing demand for medical imaging has significantly increased radiologists’ workload [17].

Self-perceived certainty in making a diagnosis, even with years of experience, may not necessarily reflect on the correctness of diagnosis. While some of the participants in our survey were independently-practicing GPs with noticeable prior experience, their responses were similarly undesirable. Other studies such as the one by Eisen et al. [12] showed a noticeable association between self-reported confidence in the diagnosis and the correctness of diagnoses for CXRs, in our study this was the case only for a minority of vignettes. The primary source of error for our participants, we believe, was the significant knowledge deficiency, which led many of them astray.

We observed an association between self-reported prior exposure to similar images and correctness of diagnosis, for some CXRs. This may show the role of education and experience for making a correct diagnosis. Given the knowledge deficiencies that our study and others [7–14] have shown, the educational curricula should focus more on areas related to acute life-threatening conditions, and optimize the time and methodologies for training proficient practitioners. A systematic approach, as well as adequate knowledge of normal anatomy, and pathologies are required by practitioners to make more accurate and timely diagnoses [18]. Of paramount importance also is to empower the practitioners to correctly diagnose a normal CXR, what they would frequently encounter.

However, it is farfetched to assume that most practitioners would get to that level of proficiency in near future. Accordingly, a pragmatic alternative is to think about timely secure ways of review of CXRs for acute pathologies by radiology experts. This could be specifically facilitated by information technology advances, such as electronic health records, or in a more simplistic way, patient-health record friendly applications (apps) that permit transmission of data elements such as images for tele-consultation via handheld devices [19, 20]. This would be particularly helpful for healthcare providers that would ultimately practice in rural or independent practices, which do not have a central radiology department staffed with a full- time radiologist.

### Limitations

Our study has several limitations. First, in our study, we used carefully selected classical vignettes and CXRs that merely had a single acute pathology. In real-world practice, patients often present with vague scenarios and CXRs might have suboptimal quality or competing pathologies. Therefore, our study has likely underestimated the proficiency limitations of practitioners, given the fact that real-world estimates of correct interpretations would likely be even lower than what we found. In that sense, our results should raise an even higher degree of concern with regards to knowledge deficiencies outlined. Second, caution should be exercised about the external validity of our findings in other countries, until future multi-centric multi-national data emerge. However, we suspect that potential knowledge deficiency would exist among non-radiology providers, albeit with some variations, around the world. Third, we noted a lower response rate among GPs compared to medical students. The reasons for this difference might be prior interactions with the study team in the academic environment, better potential understanding of the academic value of a research study thereby increasing their willingness to participate, more enthusiasm among medical students to test their own knowledge, and busier clinical schedule of the GPs. A longer time for recruiting participants might have made it possible to include more GPs, due to time constraint, however, we were unable to enroll more participants. Fourth, some other characteristics –not investigated in our analyses– can also influence readers’ performance, such as their interest in radiology or emergency medicine, extra period of time spent in radiology department voluntarily, and others. However, the overall performance of the participants was sub-optimal, with only a few participants (8 participant) who correctly diagnosed more than seven vignettes. Finally, years since graduation can impact the responses, either because of more experience over time leading into improved answers or being less fresh with the given topics leading into sub optimal answers. However, our sample of GPs was relatively small to explore such associations.

## Conclusions

In Conclusion, the diagnostic proficiency of practitioners for acute chest pathologies in our study was far from optimal, even for classic CXRs. False diagnosis in both normal and abnormal CXRs leads to inappropriate and harmful clinical decisions. To improve the quality of care for patients with acute chest diseases, the educational curricula of students, and continuous education for independent practitioners should focus more on areas related to acute life-threatening conditions. Using secure patient-information sharing platforms for immediate transfer of CXRs in acute pathologies (tele-radiology) would be a practical alternative in the interim.

## Additional files

Additional file 1: Raw data of the survey (XLSX 18 kb)

Additional file 2: Additional vignettes and associated images used in the survey (DOCX 490 kb)

## Abbreviations

ARDS: Acute respiratory distress syndrome
CXR: Chest X-ray
GP: General practitioner
SEM: Standard error of the mean

## References

[1] Pitts SR, Niska RW, Xu J, Burt CW. National Hospital Ambulatory Medical Care Survey: 2006 emergency department summary. Natl Health Stat Report. 2008;7:1–38. 18958996

[2] Hing E, Hall MJ, Xu J. National Hospital Ambulatory Medical Care Survey: 2006 outpatient department summary. Natl Health Stat Report. 2008;4:1–31. 18958995

[3] Grosvenor LJ, Verma R, O'Brien R, Entwisle JJ, Finlay D (2003). Does reporting of plain chest radiographs affect the immediate management of patients admitted to a medical assessment unit?. Clin Radiol.

[4] Lomoschitz FM, Eisenhuber E, Linnau KF, Peloschek P, Schoder M, Bankier AA (2003). Imaging of chest trauma: radiological patterns of injury and diagnostic algorithms. Eur J Radiol.

[5] Butcher BL, Nichol KL, Parenti CM (1993). High yield of chest radiography in walk-in clinic patients with chest symptoms. J Gen Intern Med.

[6] Buenger RE (1988). Five thousand acute care/emergency department chest radiographs: comparison of requisitions with radiographic findings. J Emerg Med.

[7] Satia I, Bashagha S, Bibi A, Ahmed R, Mellor S, Zaman F (2013). Assessing the accuracy and certainty in interpreting chest X-rays in the medical division. Clin Med.

[8] Christiansen JM, Gerke O, Karstoft J, Andersen PE (2014). Poor interpretation of chest X-rays by junior doctors. Dan Med J.

[9] Jeffrey DR, Goddard PR, Callaway MP, Greenwood R (2003). Chest radiograph interpretation by medical students. Clin Radiol.

[10] McLauchlan CA, Jones K, Guly HR (1997). Interpretation of trauma radiographs by junior doctors in accident and emergency departments: a cause for concern?. J Accid Emerg Med.

[11] O'Brien KE, Cannarozzi ML, Torre DM, Mechaber AJ, Durning SJ (2008). Training and assessment of CXR/basic radiology interpretation skills: results from the 2005 CDIM survey. Teach Learn Med.

[12] Eisen LA, Berger JS, Hegde A, Schneider RF (2006). Competency in chest radiography. A comparison of medical students, residents, and fellows. J Gen Intern Med.

[13] Silva VM, Luiz RR, Barreto MM, Rodrigues RS, Marchiori E (2010). Competence of senior medical students in diagnosing tuberculosis based on chest X-rays. J Bras Pneumol.

[14] Herman PG, Hessel SJ (1975). Accuracy and its relationship to experience in the interpretation of chest radiographs. Investig Radiol.

[15] Kampmeyer D, Matthes J, Herzig S (2015). Lucky guess or knowledge: a cross-sectional study using the bland and Altman analysis to compare confidence-based testing of pharmacological knowledge in 3rd and 5th year medical students. Adv Health Sci Educ Theory Pract.

[16] Wass V, Van der Vleuten C, Shatzer J, Jones R (2001). Assessment of clinical competence. Lancet.

[17] Ekpo EU, Egbe NO, Akpan BE (2015). Radiographers' performance in chest X-ray interpretation: the Nigerian experience. Br J Radiol.

[18] Raoof S, Feigin D, Sung A, Raoof S, Irugulpati L, Rosenow EC (2012). Interpretation of plain chest roentgenogram. Chest.

[19] Kammerer FJ, Hammon M, Schlechtweg PM, Uder M, Schwab SA. A web based cross-platform application for teleconsultation in radiology. J Telemed Telecare. 2015. doi:10.1177/1357633X15575237. 10.1177/1357633X15575237 25962651

[20] Rosenberg C, Langner S, Rosenberg B, Hosten N (2011). Medical and legal aspects of teleradiology in Germany. Rofo.

