# Supplementary material for: Survey of practitioners’ competency for diagnosis of acute diseases manifest on chest X-ray
Source: BMC Med Imaging. 2017 Aug 18;17:49. doi: 10.1186/s12880-017-0222-8 (PMC5563017; doi:10.1186/s12880-017-0222-8)
Supplement: Supplementary file 2 — Additional vignettes and associated images used in the survey (DOCX 490 kb) [file 12880_2017_222_MOESM2_ESM.docx]

**Additional Vignettes and Associated Images Used in the Survey:**

1. **35 year-old man presenting with acute shortness of breath and pleuritic chest pain**

Courtesy of Prof Frank Gaillard, [Radiopaedia.org](http://Radiopaedia.org), rID: [15374](https://radiopaedia.org/cases/tension-pneumothorax-8)


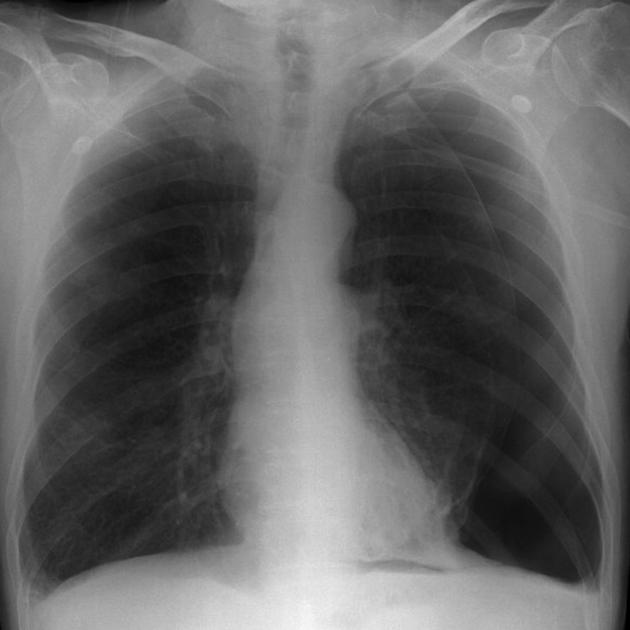


Diagnosis: **Pneumothorax**

1. **75 year-old man with end-stage renal disease but no prior heart disease presenting with gradual onset dyspnea**

Available at: <http://chestatlas.com/gallery/Pericardium/008PericeffuslargePA>


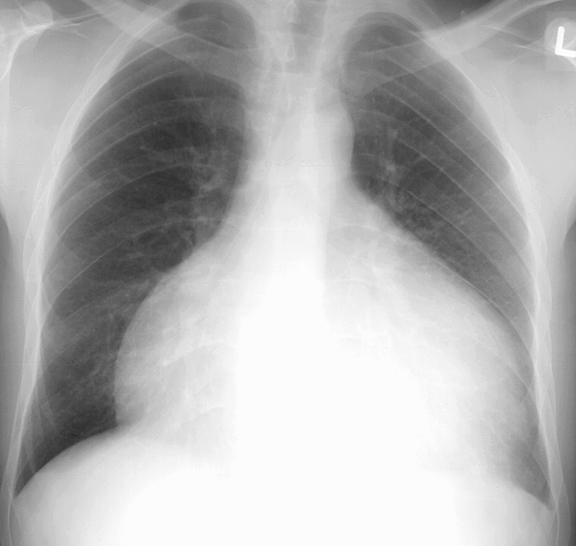


Diagnosis: **Pericardial effusion**

1. **26 year-old woman presenting with a 1-week history of intermittent minor hemoptysis**

Courtesy of Dr. Ian Bickle, [Radiopaedia.org](http://Radiopaedia.org), rID: [30197](https://radiopaedia.org/cases/inhaled-foreign-body-3)


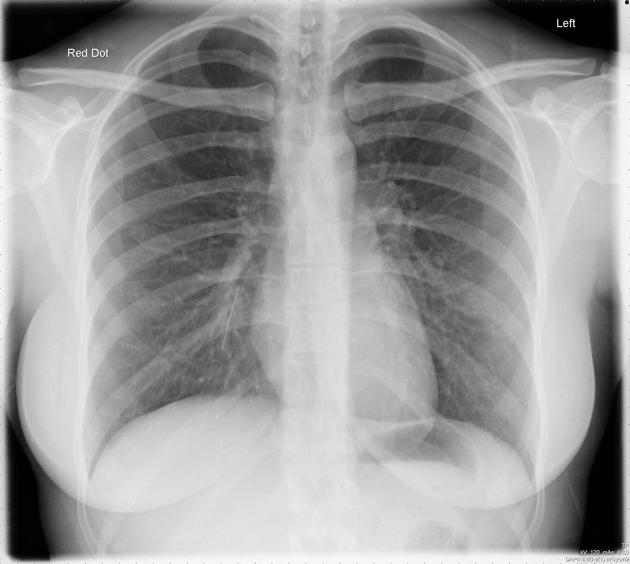


Diagnosis: **Foreign body aspiration**

1. **42 year-old man with acute abdominal pain one month after motor vehicle accident**

Available at: [http://chestatlas.com/gallery/Rupt-HD/Case_9a](http://chestatlas.com/gallery/Rupt-HD/Case_9a%20)


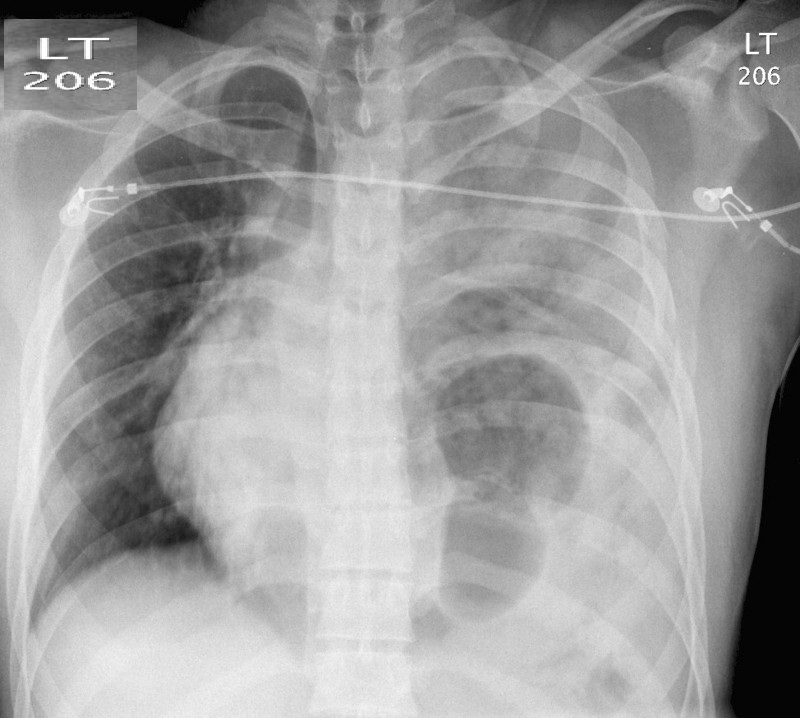


Diagnosis: **Diaphragmatic herniation**

1. **68 year-old man with a history of hypertension found hypotensive and unconscious in nursing home**

Courtesy of Dr. Hanisalam, [Radiopaedia.org](http://Radiopaedia.org), rID: [9279](https://radiopaedia.org/cases/aortic-transection)


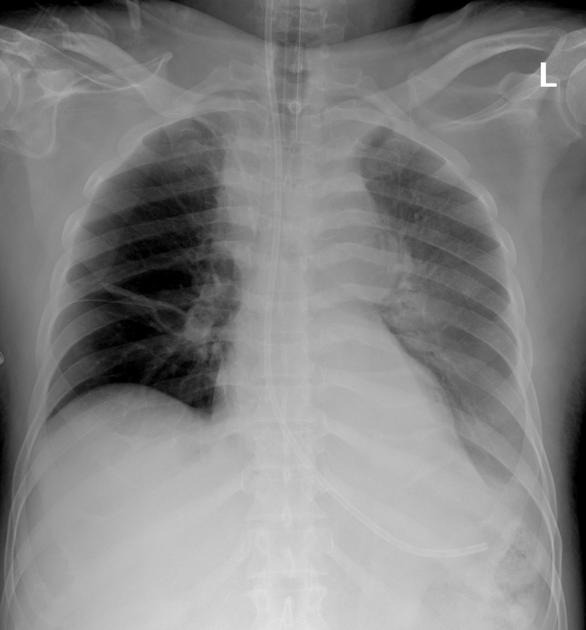


Diagnosis: **Aortic dissection**

1. **66 year-old man with severe dyspnea and hypoxemia 1 week after acute severe pancreatitis**

Patil, V. Complicated falciparum Malaria in western Maharashtra, Trop Parasitol. , 2(1), pp. 49-54. Available at: [http://www.ncbi.nlm.nih.gov/pmc/articles/PMC3593511/figure/F1/](http://www.ncbi.nlm.nih.gov/pmc/articles/PMC3593511/figure/F1/%20)


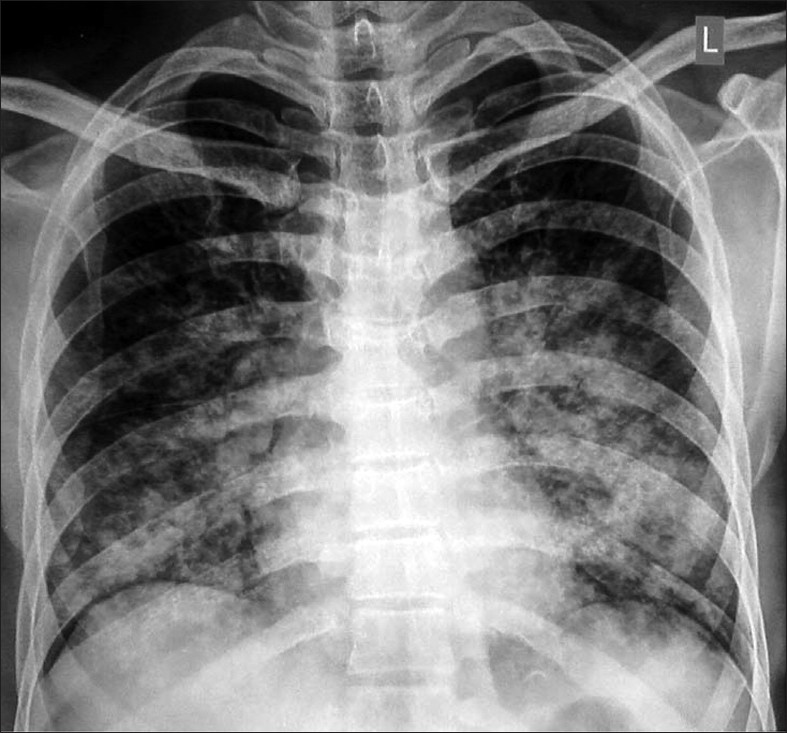


Diagnosis: **Acute respiratory distress syndrome (ARDS)**

1. **42 year-old man presenting with acute lower chest and epigastric pain**

Image available at: [http://radiologymasterclass.co.uk/tutorials/chest/chest_pathology/chest_pathology_page7.html](http://radiologymasterclass.co.uk/tutorials/chest/chest_pathology/chest_pathology_page7.html%20)

Diagnosis: Pneumoperitoneum
